# Progress and Perspectives of Desalination in China

**DOI:** 10.3390/membranes11030206

**Published:** 2021-03-15

**Authors:** Guoling Ruan, Min Wang, Zihan An, Guorong Xu, Yunhong Ge, Heli Zhao

**Affiliations:** The Institute of Seawater Desalination and Multipurpose Utilization, Ministry of Natural Resources (MNR), Tianjin 300192, China; wangmira@outlook.com (M.W.); tjdhsazh@163.com (Z.A.); labxgr@aliyun.com (G.X.); yunhongge@163.com (Y.G.); 300072@263.net (H.Z.)

**Keywords:** seawater desalination, project scale, application scenarios, standard systems, technical services

## Abstract

In recent decades, the ever-growing demands for clean water in households and industries have urged researchers to take every possible step to deal with the global water crisis. Seawater desalination has turned out to be the most promising and efficient way to provide clean water. Owing to the advancement of synthetic chemistries and technologies, great success has been achieved in the desalination and utilization of seawater worldwide. China, with the world’s largest population, has pushed the development of desalination and multipurpose utilization of seawater further in respect of materials, technologies and services, etc. This review reports recent progress of desalination technologies accomplished in China, from the viewpoints of facilities and equipment, collaborations, technologies, applications, research abilities, services, and standard systems. Inspired by the Fourteenth Five-year Plan, it also proposes future perspectives of desalination in China.

## 1. Introduction

**Figure 1:**
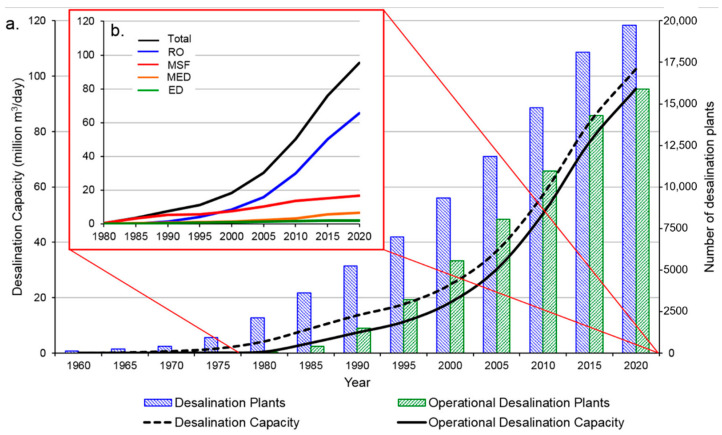
(**a**) Evolution of desalination capacity and the number of plants across the world over the past 60 years. Inset: (**b**) comparison of the desalination capacity evolution for four main desalination technologies since 1980 [3].

Reliable and stable freshwater supply is a prerequisite for the sustainable development of human society. However, the fast-growing world’s population and development of industrialization have made it difficult to acquire sufficient potable water. It has become an international task to deal with the water crisis. Freshwater has been considered as a strategic resource among the limited accessible resources [1]. Since seawater constitutes more than 97% of the total water resources, desalination offers an effective way to provide water supply via the extraction of freshwater from the sea [2]. Over the past decades, seawater desalination has evolved as the main freshwater source in many countries and areas, especially in the drought regions of the Middle East. As shown in Figure 1a, the desalination capacity has exceeded 100 million m^3^/d worldwide as the total plants number approaching 20,000 in 2020 [3].

The well-developed desalination technologies mainly include membrane-based processes (e.g., forward osmosis (FO), reverse osmosis (RO), electrodialysis (ED) and nanofiltration (NF)) and thermal-based processes (e.g., multistage flash distillation (MSF), multi-effect distillation (MED) and thermal vapour compression). Thermal processes, however, are considered to be highly energy-intensive technologies due to their reliance on thermal energy for heating the saline feed and for driving the pumps [4]. In contrast, RO has become the most commonly used technology due to its simplicity and relatively low energy cost. Since 2000, the global desalination capacity has been dominated by RO based desalination plants (Figure 1b) [3]. Till now, about 80% of the total desalination is derived from RO technology around the world [5]. It could be attributed to the advancement in novel RO membrane materials as well as the reduction in energy consumption and pure water production cost [2,6]. The energy consumption of large-scale RO and MED projects in China was estimated about 4 and 1.5 kWh/m^3^, respectively. Depending on the salinity of the feed water, RO processes can be classified into SWRO and BWRO for seawater (SW) desalination and brackish water (BW) desalination, respectively. Generally, BWRO is less energy-consuming and more efficient than SWRO because of the low feed salinity [7].

**Figure 2:**
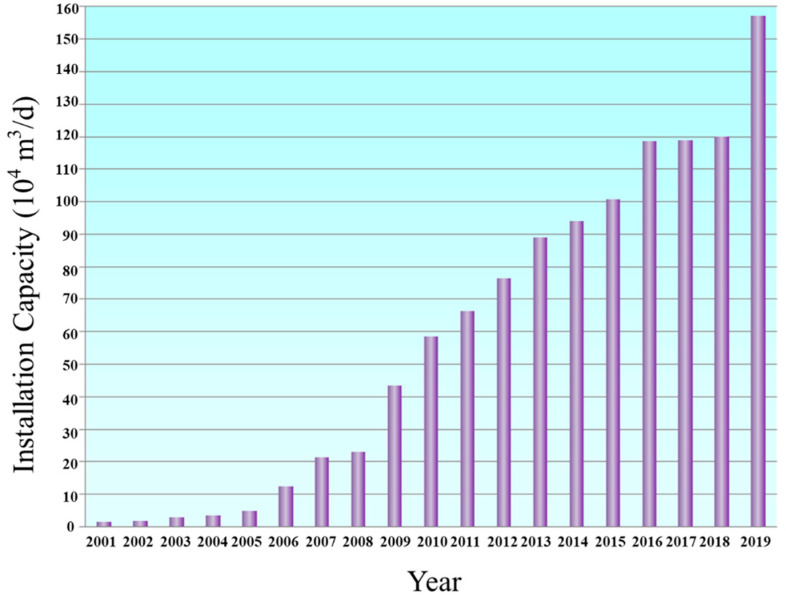
Diagram for the evolution of national seawater desalination capacity over the past two decades in China [8].

**Figure 3:**
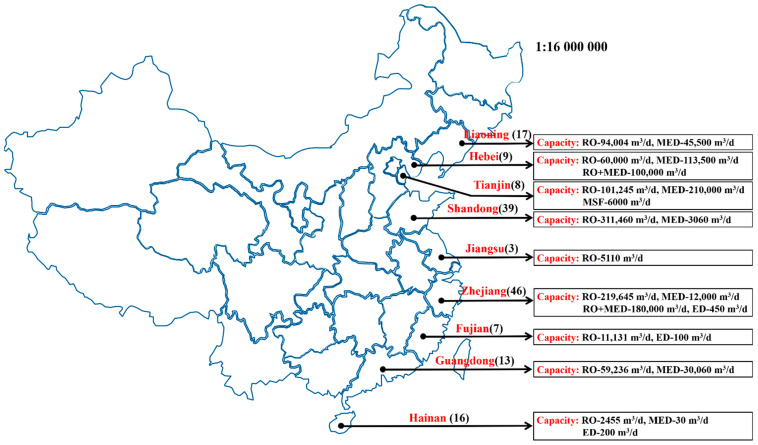
The detailed number and capacity range for local seawater desalination projects in China.

As of the end of 2019, there were 115 seawater desalination plants all together in China, with a total capacity up to 1,573,760 m^3^/d of potable water (Figure 2) [8]. Particularly, in 2019, China set up 17 desalination plants in Liaoning, Hebei, Shandong, Jiangsu, and Zhejiang, respectively, enabling a desalination capacity of about 399,055 m^3^/d. These plants were mainly built to supply the coastal industries, including petrochemical industries, steel industries, fossil plants and nuclear plants, etc. The detailed number and capacity range for local seawater desalination projects in China is depicted in Figure 3.

Of all the seawater desalination plants in China, a majority of 97 plants are operated based on RO, possessing a capacity of 1,000,930 m^3^/d, which accounts for 63.6% of the total capacity. The second most popular type is MED plants, the number of which adds up to 15, generating 565,530 m^3^/d water desalination capacity, about 35.9% of the total capacity. There is only one plant that applies the MSF technique, with the capacity of 6000 m^3^/d, in the proportion of 0.38% of the total capacity. Three other plants rely on electrodialysis technique, exhibiting a capacity of 800 m^3^/d, less than 0.05% of the total capacity. Another one is based on FO technique, providing a desalination capacity of 500 m^3^/d, about 0.03% of the total capacity. Over several decades’ evolution, China has gained great progress in desalination industries in terms of products and engineering procurement construction (EPC) companies (Table 1).

So far, there have been many interesting review papers on the seawater desalination technology and engineering in China, ranging from the desalination industries, techniques, projects, and energy consumption to economic impact, etc. [9,10,11,12,13,14]. A comprehensive review covering the recent project scales, application scenarios, equipment series, standard systems and technical services undergoing in China, especially those initiated by the leading desalination organization of the Institute of Seawater Desalination and Multipurpose Utilization (ISDMU) is still missing. Therefore, in this review, the recent progress on desalination projects and techniques achieved by China are surveyed. Emphasis is put on the project scale, application scenarios, equipment, techniques, materials and services, especially achieved by ISDMU. In the end, it comes up with some perspectives in the future development of desalination in China.

## 2. The Main Technical Progress

### 2.1. The Expanded Project Scale and Single Plant Scale

**Figure 4:**
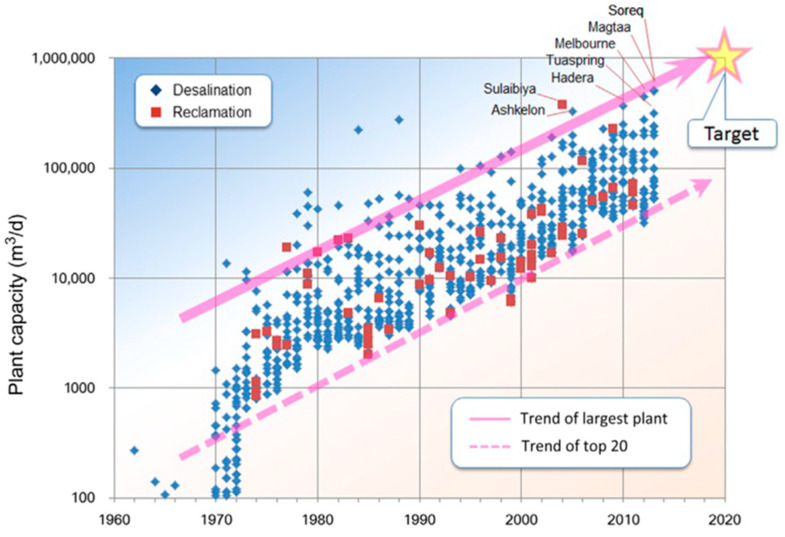
The evolution of plant capacity worldwide over the past 60 years. The solid line shows the trend of the capacity of the largest plant and the dashed line showing that of the top 20 plants [15].

Plant capacity is one of the key indicators of the development degree of the desalination industry. To scale up the plant facility and capacity is highly desirable as larger plants offer the distinct advantages of energy-effectiveness, operational stability, and small footprint. The last 60 years have witnessed the evolution of the seawater desalination capacities of the top 20 plants worldwide, as shown in Figure 4 [15]. All of the plants had undergone steady increases in the plant capacities, especially the largest one in Sorek in Israel, the capacity of which increased by almost 1000-fold over 60 years (achieved 624,000 m^3^/day) [16]. It is predicted that the capacity of a single SWRO plant could exceed 1,000,000 m^3^/d in the future.

In recent years, China has been devoted to expanding the scale of plants as well. So far, the ≥10,000 m^3^/d desalination plants have added up to 37, with a total capacity of 1,403,848 m^3^/d. The number of plants with the capacity in therange of 1000–10,000 m^3^/d has reached 42, exhibiting a capacity of 162,522 m^3^/d. There are 36 plants under 1000 m^3^/d, with the total capacity of 7390 m^3^/d. In 2019, the capacity of largest newly built desalination plant is 180,000 m^3^/d.

**Figure 5:**
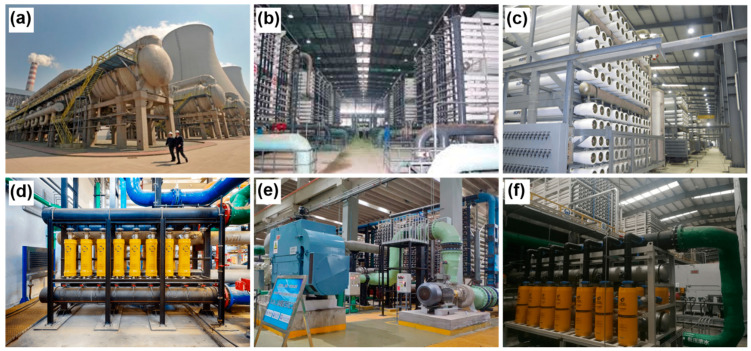
Seawater desalination projects with the capacity of >50,000 m^3^/d in China. (**a**) Beijiang Power Plant MED project, Tianjin (200,000 m^3^/d). (**b**) Dagang New Spring SWRO project, Tianjin (100,000 m^3^/d). (**c**) Caofeidian SWRO project, Tangshan (50,000 m^3^/d). (**d**) Befesa SWRO project, Qingdao (100,000 m^3^/d). (**e**) Dongjiakou SWRO project, Qingdao (100,000 m^3^/d). (**f**) Zhejiang Petroleum SWRO/MED project, Zhoushan (180,000 m^3^/d).

The representative seawater desalination projects with the capacity of >100,000 m^3^/d are shown in Figure 5. In China, the largest seawater desalination plant of the Beijiang Power Plant MED project owns a capacity of about 2,000,000 m^3^/d (Figure 5a), whilst the largest SWRO plants in five corresponding projects have the capacity of 100,000 m^3^/d each (Figure 5b–f). Notably, Befesa SWRO project is attempting to expand its capacity to 200,000 m^3^/d in the expectation of becoming the largest SWRO plant in China soon. Moreover, it has become more and more popular to apply the integrated membrane/distillation systems in large plants. For example, in Zhejiang petroleum project, it makes good use of SWRO (75,000 m^3^/d) and MED (105,000 m^3^/d) in the integrated system.

### 2.2. The Broadening Application Scenarios and Technical Integration

**Figure 6:**
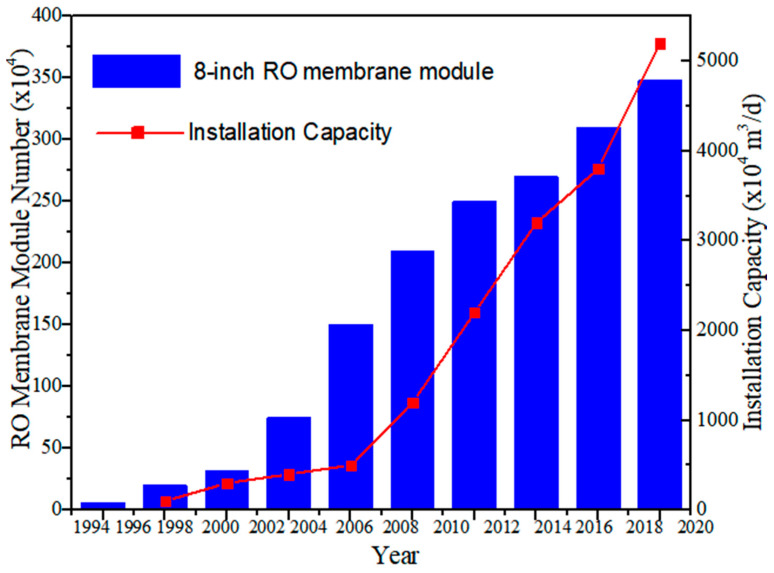
The holding number of RO membrane modules and the installation capacity in China.

Apart from seawater desalination, desalination technologies have been widely used in other areas, such as water treatment for municipal and industrial usages. According to the statistic released by The Membrane Industry of Association of China (MIAC), the holding number of RO membrane modules has exceeded 3,500,000 and the installation capacity outnumbered 50,000,000 m^3^/d (Figure 6) [17]. Among them, only about 3% of the capacity was provided to seawater desalination projects (1573,760 m^3^/d), indicating that RO membranes have been extensively used in many other water treatment projects. Moreover, combination of different desalination technologies has become a new trend for various water treatment scenarios [18,19,20]. For instance, in the project of coal chemical industrial wastewater treatment, RO was coupled with membrane bioreactor (MBR) and MED processes as an integrated system, providing a capacity of 20,000 m^3^/d.

**Figure 7:**
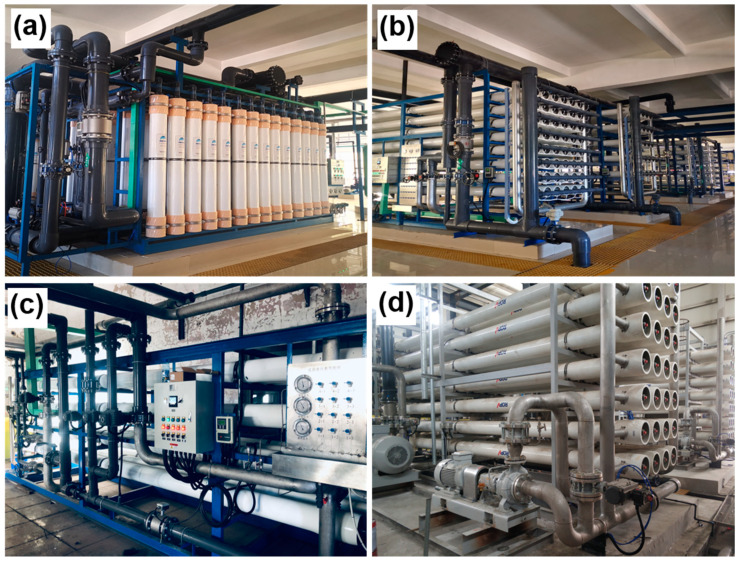
Desalination technologies used in industrial wastewater treatment projects from ISDMU. The ultrafiltration (**a**) and RO (**b**) systems for Lianyuan power plant project, Neimeng. (**c**) Temier power plant project, Neimeng. (**d**) Tianyu coal chemical industry project, Xinjiang.

The ISDMU of the Chinese Ministry of Natural Resources, has taken over several industrial wastewater treatment projects in recent years (Figure 7). Despite the application in seawater desalination, desalination technologies have been applied in a wide range of water treatment applications.

**Figure 8:**
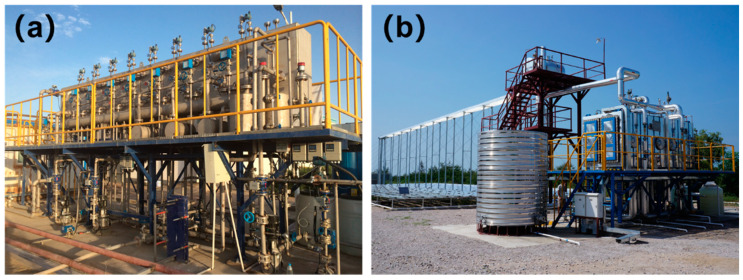
Solar energy driven desalination projects in China. (**a**) Solar-driven eight-effect plate distillation in Luntai, Xinjiang (MEP, 24 m^3^/d). (**b**) Solar-driven low temperature MED in Ledong, Hainan (30 m^3^/d).

On another hand, efforts have been made in the combination of desalination with other energy technologies, such as nuclear power. Seawater desalination is an energy-intensive and/or thermal-intensive process, while nuclear power plants generally rely on excessive consumption of water for cooling and other operations [21]. The integration of seawater desalination and nuclear power plants could be beneficial for each other. Hongyanhe nuclear power plant SWRO project (10,000 m^3^/d) is the first to demonstrate the practical application of nuclear power and desalination integrated system. After that, SWRO desalination projects have been utilized in other nuclear power plants, such as Ningde, Sanmen, and Haiyang. Recently, researchers from Shandong have proposed an innovative integration idea, named “Co-transport of water and heat”. In their proposal, the product water from the nuclear desalination undergoes certain post-treatment processes first, following heating by the extra heat from the nuclear power plant. Then the heated desalination water was transported to the users, providing heat and clean water simultaneously. Using this proposal, the traditional three pipe systems could be replaced by one single pipe, thereby reducing the operation cost remarkably. The pilot project for water and heat simultaneous transmission has been put into operation, providing water and heat supply for nearly 2000 people at the same time. It provides technical support and demonstration for long-distance heat and water transmission. Additionally, other renewable energy sources such as solar, wind, and geothermal energy have also been considered as an alternative energy supply or desalination [22]. Particularly, solar energy has been widely applied as a heat source or power source for thermal and/or membrane desalination. In China, several solar-driven desalination projects have been implemented. For example, ISDMU has constructed a solar-driven eight-effect plate distillation in Xinjiang (Figure 8a) and solar-driven MED in Hainan (Figure 8b).

### 2.3. Further Improved Equipment Series and Strengthened Supporting Capability

Recent decades have seen great progress and advances in the evolution of seawater desalination facilities and capabilities for marine use. Particularly, ISDMU has developed the abilities in equipment series (Table 2) supply based on different utility sites and energy sources, which are classified as ship-based equipment, island-based equipment and all-in-one integrated equipment.

**Figure 9:**
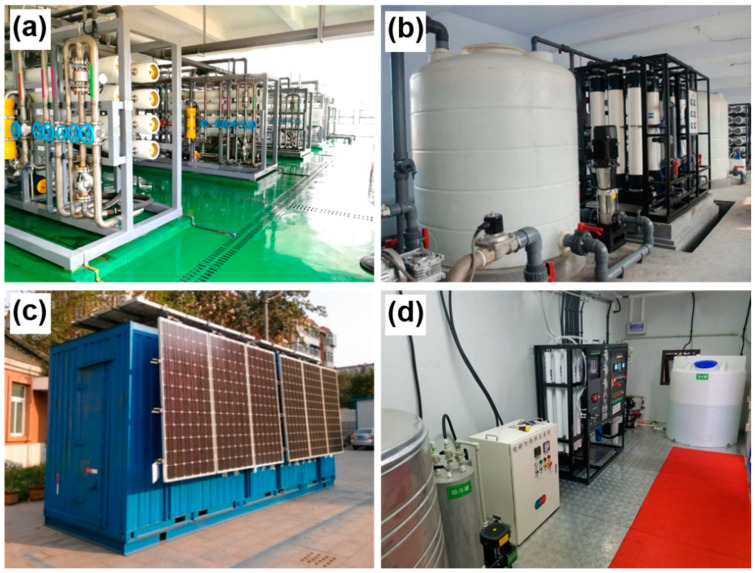
Photographs of some different desalination devices from ISDMU. (**a**) Yongxing (capacity, 1000 m^3^/d). (**b**) Lingshan (capacity, 300 m^3^/d). (**c**) The all-in-one system (wind, solar, oil, desalination, and intelligent control integrated into one system). (**d**) The South Pole (the capacity of 5 m^3^/d).

Generally, island-based seawater desalination units are located at remote islands and marine monitoring stations. These instruments should have the advantages of compact structures, less area occupation, easy to install, high automation, easy to operate and maintain. Examples include equipment series in Yongxing island (Figure 9a, with a capacity of 1000 and 100 m^3^/d, respectively), Chenhang island, Lingyang island, Dongmaozhou island, Xiaoqin island and Zhongjian island (with a capacity of 100 m^3^/d), Lingshan island in Qingdao (Figure 9b, with a capacity of 300 m^3^/d), and Daguan island in Qingdao (with a capacity of 5 m^3^/d using renewable energy). Alternatively, an all-in-one integrated system has been designed and developed to supply fresh water for remote places with no power or network coverages. With the combination of wind, solar, oil, desalination, and intelligent control systems, such a highly integrated plant has its energy supply systems without the need of additional power resources, thereby showing the advantageous features of energy-effective and highly automated operation (Figure 9c). Moreover, a low-temperature tolerable RO equipment realized by integration with thermal devices for extreme conditions, like in the South Pole (Figure 9d), was developed.

**Figure 10:**
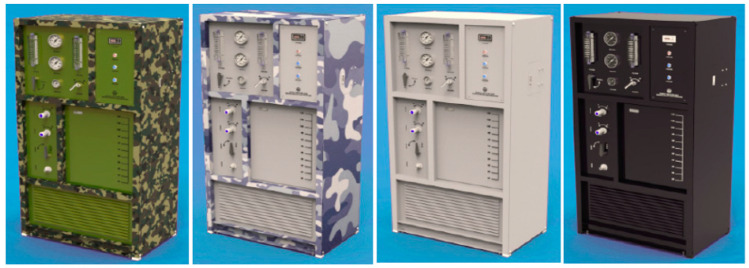
Seawater desalination devices for ship use [23].

Besides, seawater desalination has been extensively employed to generate freshwater to meet the regular need onboard ships (Figure 10) [23]. Such devices are designed specifically for ship users, offering the advantages of small size, lightweight, simple installation, and wide adaptability. Plus, the maintenance of these instruments is convenient and time-saving without frequent washing, as the instruments use one-key start/stop allowing the installation/maintenance to be carried out in narrow spaces, such as the cabins, decks, and corridors [23].

ISDMU has also developed chemical reagents for seawater desalination including scale inhibitors, defoaming agents, fungicides, and cleaning agents [24]. Scale inhibitors showed excellent performance, with the antiscale rate at about 95% [24]. The defoaming agents composed of polyether (SD501) and silicone (SD502), exhibited a defoaming rate of >90% at 70 °C without interfering with the scale inhibitors [24]. The developed DM fungicides have the advantages of broad-spectrum and fast sterilization, easy degradation and residual-free. Meanwhile, the cleaning agents are safe, environmentally friendly, as well as compatible with scale inhibitors and fungicide used. These new reagents have been applied in many desalination projects such as Baifa, Yongxing island, Cape Verde, and Djibouti seawater desalination plant.

In the typical SWRO system, pretreatment processes are utilized to ensure all the particulates are removed before the stream reaching the membranes without the incorporation of any strainers [5,25,26]. However, such pretreatment process is sensitive to the changes of source water characteristics, which would lead to a large consumption of processing chemicals, high operational costs and deterioration of environment [27,28]. Thus, it is desired to come up with new processes that allow minimum consumption of chemicals in different application scenarios.

### 2.4. Improved Research and Development Ability and Technical Level

#### 2.4.1. Core Components and Equipment

**Figure 11:**
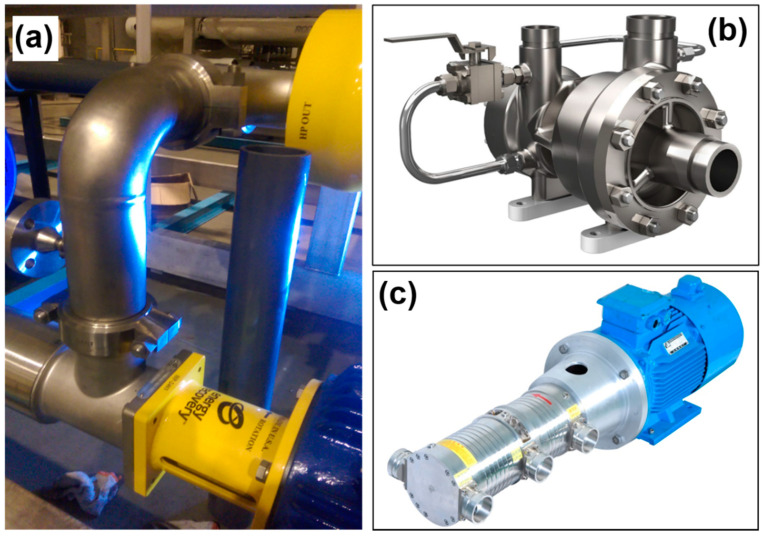
Photographs of (**a**) titanium-based pipelines and (**b**,**c**) high-pressure pumps.

Despite the rapid development of seawater desalination technologies in China, the core materials, components, and equipment (e.g., RO membranes, energy recovery devices, and high-pressure pumps) are still cost sensitive on the importation. In recent years, ISDMU has been devoted to the research and development of key devices of desalination based on theoretic improvement, self-developed simulation and domestic materials. For instance, ISDMU developed a series of titanium-based pipelines and its fittings (Figure 11a), which showed excellent performances in several tropical island seawater desalination projects, especially the anticorrosive activity [29]. Energy recovery devices and high-pressure pumps were developed with higher efficiency and recovery tolerance (Figure 11b,c) [30,31]. The experiences of high-pressure components and the data accumulation of its site applications and cost evaluation will set up new frontier for SWRO material selection.

#### 2.4.2. Membrane Materials and Technologies

During a desalination process, the membrane is one of the key elements determining the desalination performance. Generally, there are two main types of desalination membranes, namely ultrafiltration (UF) and RO membranes. However, membrane fouling (e.g., organic fouling, scaling/inorganic fouling, and biofouling) is a major concern in membrane-based applications [32]. For example, scaling could induce extra energy and operational cost in the RO process as a result from the contamination in feed water [33]. Membrane surface modification has been developed to improve the membrane antifouling properties, by physical or chemical methods like nanomaterial penetration, mixing, coating, grafting, self-assembly, chemical coupling, physical adsorption, and irradiation, etc. [32,34,35,36,37].

**Figure 12:**
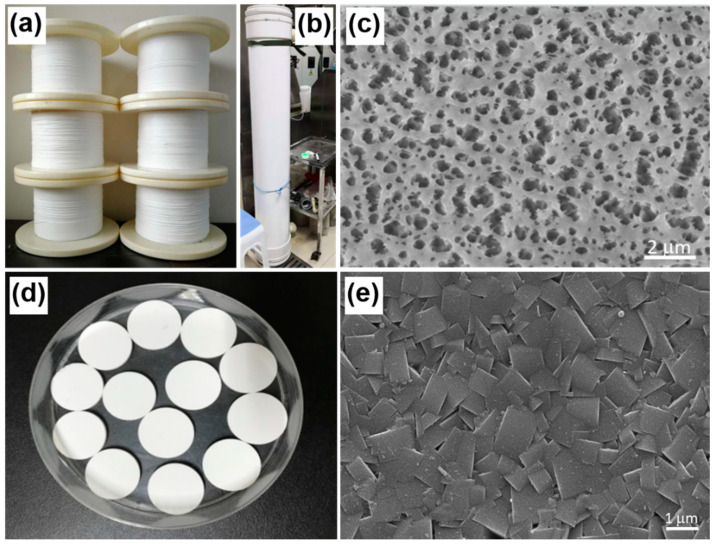
Photographs of (**a**) PTFE hollow-fiber membrane and (**b**) the membrane module. (**c**) Scanning electron microscopy (SEM) surface image of the PTFE hollow-fiber membrane. (**d**) Photograph and (**e**) SEM surface image of the MFI-type zeolite membrane (unpublished work).

On another hand, novel membrane material synthesis, module design and processing approaches have been broadly explored, rendering the membranes chlorine-resistant, antifouling, stable, and highly efficient in terms of desalination and separation performance. Interested readers are referred to the excellent research and review articles [38,39,40,41,42,43,44,45,46]. In a recent work by ISDMU and Shandong University of Science and Technology, aminophenyl-modified mesoporous silica NPs were used to fabricate RO membranes, enabling enhanced water flux by 21% while maintaining high NaCl rejection of 98.97%, only slightly decreased by 0.29% compared with that of the pristine membrane [47]. Polytetrafluoroethylene (PTFE) hollow-fiber has emerged as a promising candidate for highly efficient water treatment applications due to its acid-resistant, alkali-resistant and antioxidant properties. Recently, ISDMU has developed a pilot line for PTFE hollow-fiber membranes (HFMs), producing versatile PTFE HFMs with tunable diameters of 0.8–1.5 mm and average pore size 0.28–2.17 µm (Figure 12a,c). The self-developed 10-inch module (Figure 12b) based on the produced PTFE HFMs yields the capacity of portable water of 140 L/h with a salt rejection of 99.9% in MD desalination. Apart from MD, PTFE HFMs are also widely used for UF desalination. Alternatively, ceramic membranes have been developed with outstanding features of tunable microstructures, excellent chemical and thermal properties, long lifetime, and little environmental impact [48]. Ceramic membranes are mostly used in UF, whilst in some cases, are used in NF and RO desalination applications. Some new types of ceramic membranes made of low-cost geomaterials from nature (i.e., clay, apatite, zeolite, and sand) have become a focus of research interests [49,50,51,52]. For instance, ISDMU has invented novel MFI-type zeolite membranes, for RO desalination applications (Figure 12d,e, unpublished work), exhibiting high salt rejection with diminished absorption for hydrated metal ions in saline water.

Researchers from the Nanjing University of Technology have promoted the advancement of ceramic membranes and cut the price of imported ceramic membranes in Chinese markets [17]. It is mainly realized by shifting the research focus from process engineering to nanostructured membrane design, the fabrication technology from experience-driven to quantification control. Up to now, the domestic enterprise values of the ceramic membrane industries have risen from the initial million to 100-million scale. Large scale production lines for ceramic membranes have enabled their applications in traditional Chinese medicine clarification, biological fermentation broth purification, petro-chemical as well as environmental protection fields [17]. The ceramic membranes developed in China exhibited better separation and operational stability, more than 1000 application cases, and exported to 55 countries. Some typical ceramic membrane projects have been listed in Table 3. In supplement for ultra-large companies, listed companies, large private enterprises, as well as research institutions, the ceramic membrane markets have brought about more than one-billion-yuan direct profits and 10-billion-yuan indirect profits [17]. The development of ceramic membranes in Chinese industries is considered to be continuously accelerating in the future.

#### 2.4.3. Chemical Resources Recovery

Seawater contains a large number of chemicals which could potentially be extracted to add to the chemical resources on the land. It has drawn the world’s attention to recover and reuse these chemical resources for the sake of sustainable development of natural resources. In conventional desalination process, especially SWRO, rejected brine containing the majority of chemicals was supposed to discharge into the ground or sea. Thus, it is essential to improve the recovery of chemical resources from the brine to alleviate the discharge environmental compact. Various strategies have been proposed, including solar ponds, membrane distillation/crystallization, electrodialysis and reverse electrodialysis, chemical precipitation, adsorption/desorption, eutectic freezing and crystallization, pressure infiltration and microbial desalination ponds [53]. Apart from salt, the main chemical resources produced from brine include bromine, potassium chloride, magnesium chloride, magnesium sulfate, and potassium sulfate, etc. In China, most of such enterprises are located in Tianjin, Hebei, Shandong, Fujian, and Hainan. The Chinese government offers great financial supports for research programs on chemical resource recovery from brine, promoting advances in unique chemical extraction technologies, high efficiency equipment and industrial-level energy-saving demonstration. ISDMU has achieved progress in the research of continuous hydrothermal preparation of high purity magnesium hydroxide as well as in the construction of a pilot line for the macro process. Still, there are some challenges in these areas such as high costs, large energy consumption, relatively deficient equipment, and low efficiency. In 2019, Tianjin Changlu Hangu Saltern Co., Ltd. (Tianjin, China) successfully implemented the equipment remodeling for bromine recovery from highly concentrated seawater, enabling the construction of thousand-ton demonstration installation. As a result, the energy consumption was reduced by nearly 10% and the recovery rate increased by 8%, providing excellent demonstration for further applications of this technology in Shandong and other places (unpublished work). The extraction of strategic chemical elements such as lithium and uranium through inorganic ion adsorption method is still in the experimental stage.

#### 2.4.4. Boron Removal

Seawater contains varied content of boron in different geographical locations. Typical, the boron content is in the range of 4–6 mg/L in most seawater resources whilst about 4.6 mg/L in standard seawater [54]. The existence of residual borides from desalination has become a major concern among people, as minute boron would be detrimental to human health and plant growth. The limit level of boron content in drinking water was established in different countries to guarantee safe drinking, for example, the World Health Organization (WHO, 2.4 mg/L, 2011), Japan (1.0 mg/L, 2015), European Commission (1.0 mg/L, 1998), and China (0.5 mg/L, 2006) [55]. As the majority of the boron compounds in seawater is in the form of small molecules of H_3_BO_3_ and the remaining being H_2_BO_3_^-^, it is difficult to get rid of boron residues to meet such criterion via traditional desalination [55]. In fact, the removal of boron can be sensitive to the temperature, salinity and pH of the feed water in the thermal desalination processes [56]. Whilst, in SWRO, the boron removal level is closely related to the pH value, which affects the dissociation of boric acid and surface charge negativity of the RO membrane [57]. Moreover, two-pass system or integrated technologies have been adopted in SWRO to realize the higher boron removal in desalination [58].

#### 2.4.5. Novel Technologies

**Figure 13:**
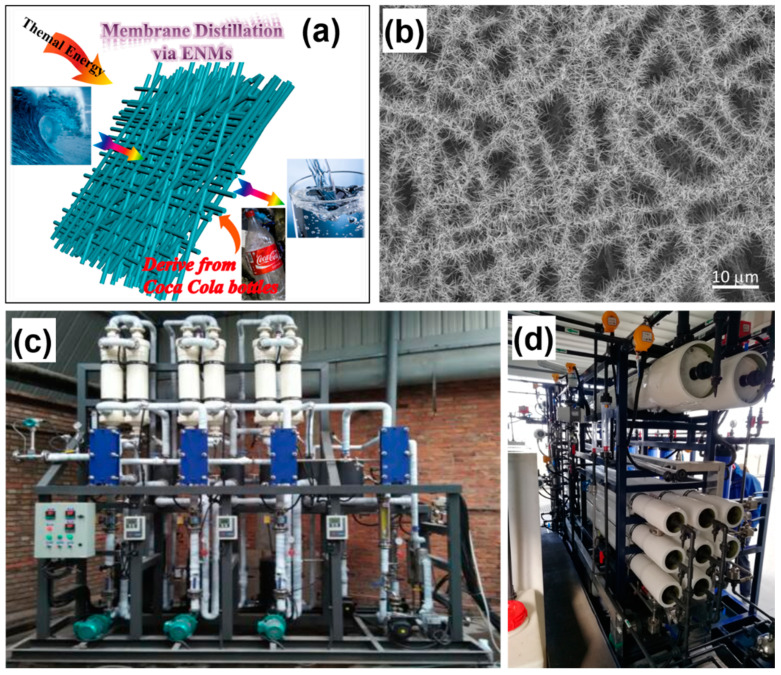
(**a**) A brief illustration of MD membranes based on the electrospun nanofibrous membranes derived from Coca Cola bottles [59]. (**b**) SEM image of the hierarchical nanostructures on nanofibrous membranes (unpublished work). (**c**) Three-effect MD pilot equipment with a capacity of 2 m^3^/d. (**d**) A hybrid FO-MD system.

ISDMU has undertaken a lot of research based on desalination technologies beyond traditional SWRO process, including but not limited to MD, FO and CDI [59,60,61]. For example, Xu et al. reported a novel electrospun nanofibril membrane derived from Coca Cola bottles (Figure 13a), which was successfully used in the MD process [59]. In another ongoing work, the performance of the membrane was improved by enhancing the hydrophobicity of the membrane surface through hierarchical inorganic nanostructure design (Figure 13b). Figure 13c displays the pilot equipment based on a novel three-effect MD process, the capacity of which turned out to be 2 m^3^/d. This configuration was designed to promote thermal energy efficiency by reusing latent evaporation heat. It should be noted that FO, MD, or CDI is not standalone and always combined with other technologies (i.e., hybrid configuration) when being used in desalination [62]. For example, FO-NF, FO-RO, and FO-MD hybrid systems were designed for wastewater reuse [63,64,65]. A FO-RO hybrid system was used to improve the antifouling and antiscaling properties of desalination [66]. A RO-MD-PRO hybrid system was used to increase the energy efficiency of desalination [67]. In China, FO and MD have been applied in high-concentration water treatment whilst CDI is only for lab-scale study. As depicted in Figure 13d, an FO-MD hybrid system was designed exhibiting a capacity of 12.5 m^3^/d. FO, as a spontaneous osmotic-driven process, was coupled with MD to recover water from draw solutions while the MD process was used to concentrate the draw solution from FO to obtain the maximum energy utilization. Furthermore, as an emerging novel desalination technology, FO has undergone significant development during the past decades, due to its high energy efficiency and favorable separation performances [68]. Interested readers are referred to the excellent review literature on FO [69,70,71].

### 2.5. Enriched Standard Systems and Technical Services

As of the end of 2019, China had established 166 standards on seawater utilization, including 43 national standards, 116 industrial standards, and 7 local standards. ISDMU is conducting the first ISO standard on the product water of seawater desalination and it is speculated to be released in 2021.

ISDMU is planning to build the seawater desalination innovation base, combining the functions of scientific research, detection and evaluation, product development, reconnaissance design, communication and training, and information integration. In November of 2016, the construction of pilot laboratory started, with the investment of about CNY 0.46 billion for infrastructure and about CNY 0.5 billion for instruments and equipment. This pilot laboratory would provide platforms for seawater resources innovative utilization, including seawater desalination innovative service, island and vessel use small-medium desalination manufacturing, preparation and evaluation of chemical agents, and desalination device monitoring/evaluating platforms. The second phase project would mainly involve the construction of research buildings, testing centers, information centers as well as reconnaissance and design institute, etc.

This project would bring about a series of benefits for the desalination market and industry which entail (1) providing high-end research facilities for breaking the bottleneck technical problems for self-developed products; (2) enabling the third-party evaluation of related products from home and abroad and promoting their market application; (3) offering industry–academia–research innovative centers and attracting excellent research teams from both home and abroad for collaborations; (4) enabling the pilot experiments of the laboratory outcome to facilitate the maturation of the technology; (5) attracting the joining of innovative technologies and personnel to become the center of innovation and one of the world’s competitive desalination industrial communities

## 3. Summary and Perspectives

In the long term, it is of significant importance to develop desalination technologies to face the challenges of global water crisis. Desalination technologies in China are vital both in the field of freshwater extraction from the sea and the improvement of water environment and ecology. Specifically, emphasis should be put on the following aspects.

(1) Technology innovation

Further improve the performance of large-scale SWRO plants. Efforts include the enlargement of single unit capacity, decrease of energy consumption, improvement of system integration, operational stability and reliability, and desalination cost. Further endeavors should be made towards pretreatment and system instrumentation, high-performance RO membranes and elements, high-pressure pumps and energy recovery devices, etc.

(2) Utilization of chemical resources from seawater

The extraction and reuse of chemical resources from seawater desalination is an attractive topic from a scientific point of view. Better technologies need to be explored to recover chemicals and reduce possible cost. Moreover, the extraction of strategic elements such as lithium and uranium is more challenging and needs better technologies.

(3) Green pretreatment methods

Attention should be paid to the development of green pretreatments involving green antiscaling agents and agent-free biological methods. It is advisable to investigate electrocoagulation and dissolved air flotation techniques.

(4) Emerging desalination technologies

Apart from the traditional SWRO desalination technologies, emerging technologies including MD, CDI, and ED desalination processes require more research efforts, from the exploitation of novel membrane materials to the fabrication of core components and equipment. In addition, hybrid systems coupling the traditional technologies and the emerging technologies should be further promoted to make the most of the less-popular technologies.

Overall, with the ongoing research and development of desalination, China is believed to play a more and more important role in the international desalination market with remarkable openness and inclusiveness, providing state-of-the-art desalination technologies, facilities and services, benefitting the water-stressed countries and regions in the world.

## Figures and Tables

**Table 1:** Main products and EPC companies in China.

Classification	Product Brands/Company Names	Location
Membranes	OriginWater	Beijing
	Vontron	Guiyang
	Toray Bluestar	Beijing
	Koch	Beijing
	Zhaojin Motian	Yantai
	Motech	Tianjin
	Scinor	Beijing
Equipment	Shandong Shuanglun	Weihai
	Nanfang Pump Industry	Hangzhou
	Voitu	Shanghai
	Kaiquan	Shanghai
	Sulzer (Dalian)	Dalian
	KSB(Shanghai)	Shanghai
EPC company	Beijing OriginWater Technology Co., Ltd.	Beijing
	Qingdao Water Group Co., Ltd.	Qingdao
	Shanghai Electric Group Co., Ltd.	Shanghai
	ISDMU	Tianjin
	Hangzhou Water Treatment Technology Development Center Co., Ltd.	Hangzhou
	POWCHINA	Beijing

**Table 2:** Specifications of equipment models from ISDMU.

Model	Water Production Capacity (m^3^/d)	Salt Rejection (%)	Recovery Ratio (%)	Operational Pressure (MPa)	Equipment Size L × W × H/cm
CH-001 ^a^	1	99.2	12	5.5	100 × 60 × 150
CH-005	5	99.2	24	5.5	100 × 60 × 150
CH-010	10	99.5	30	5.5	100 × 60 × 160
CH-020	20	99.5	30	5.5	150 × 75 × 180
CH-050	50	99.5	40	5.5	190 × 110 × 180
CH-100	100	99.5	40	5.5	220 × 130 × 180
CH-200	200	99.5	40	5.5	400 × 150 × 180
DH-005 ^b^	5	99.5	24	5.5	100 × 60 × 150
DH-010	10	99.5	30	5.5	100 × 60 × 160
DH-020	20	99.5	30	5.5	100 × 60 × 160
DH-050	50	99.5	40	5.5	120 × 60 × 170
DH-100	100	99.5	40	5.5	120 × 60 × 170
DH-200	200	99.5	40	5.5	150 × 60 × 180
DH-500	500	99.5	40	5.5	200 × 60 × 180
DHP-005 ^c^	5	99.5	30	5.5	600 × 280
DHP-010	10	99.5	30	5.5	1000 × 300
DHP-020	20	99.5	30	5.5	1300 × 300
DHP-050	50	99.5	40	5.5	2000 × 400
DHP-100	100	99.5	40	5.5	2500 × 500

Note: ^a^ CH model series refers to desalination plant for marine use. ^b^ DH model series refers to desalination plant for island use. ^c^ DHP series refers to wind/light/oil-storage-integrated desalination plant.

**Table 3:** Summary of some typical ceramic membrane projects in China.

Company	Project Name	Area	Feed Solution	Capacity
Jiangsu Jindong Salt Refining Co., Ltd.	Salt refining project	Process dissociation	Saline water	800 m^3^/h
Wudi Xinyue Chemical Co., Ltd.	Ionic membrane caustic soda one pass salt refining project	Process dissociation	Saline water	360 m^3^/h
Shandong Haobang Chemical Co., Ltd.	Ionic membrane caustic soda one pass salt refining project	Process dissociation	Saline water	360 m^3^/h
Hulun Buir Northeast Bufeng Biotechnology Co., Ltd.	Continuous separation of fermented broth	Process dissociation	Fermented broth	1200 m^3^/d
Xinjiang Bufeng Biotechnology Co., Ltd.	Continuous separation of fermented broth	Process dissociation	Fermented broth	Valine, 900 m^3^/d Isoleucine, 800 m^3^/d
Yili Chuanning Biotechnology Co., Ltd.	Continuous separation of fermented broth	Process dissociation	Fermented broth	400 m^3^/d
North China Pharmaceutical Hebei Huamin Company, Ltd.	The continuous filtration system of fermented broth	Process dissociation	Fermented broth	300 m^3^/d
